# The association between ambient pollutants and influenza transmissibility: A nationwide study involving 30 provinces in China

**DOI:** 10.1111/irv.13177

**Published:** 2023-07-23

**Authors:** Jiao Yang, Guohui Fan, Li Zhang, Ting Zhang, Yunshao Xu, Luzhao Feng, Weizhong Yang

**Affiliations:** ^1^ School of Population Medicine and Public Health Chinese Academy of Medical Sciences & Peking Union Medical College Beijing China; ^2^ National Center for Respiratory Medicine National Clinical Research Center for Respiratory Diseases, China‐Japan Friendship Hospital Beijing China; ^3^ Institute of Respiratory Medicine Chinese Academy of Medical Sciences & Peking Union Medical College Beijing China; ^4^ Department of Clinical Research and Data management Center of Respiratory Medicine, China‐Japan Friendship Hospital Beijing China; ^5^ School of Life Course and Population Sciences King's College London London UK

**Keywords:** ambient pollutants, influenza, transmissibility

## Abstract

**Background:**

The impact of exposure to ambient pollutants on influenza transmissibility is poorly understood. We aim to examine the associations of six ambient pollutants with influenza transmissibility in China and assess the effect of the depletion of susceptibles.

**Methods:**

Provincial‐level surveillance data on weekly influenza‐like illness (ILI) incidence and viral activity were utilized to estimate the instantaneous reproduction number (R_t_) using spline functions. Log‐linear regression and the distributed lag non‐linear model (DLNM) were employed to investigate the effects of ambient pollutants—ozone (O_3_), particulate matter ≤2.5 μm (PM_2.5_), particulate matter ≤10 μm (PM_10_), nitrogen dioxide (NO_2_), sulfur dioxide (SO_2_), and carbon monoxide (CO)—on influenza transmissibility across 30 Chinese provinces from 2014 to 2019. Additionally, the potential effects of the depletion of susceptibles and regional characteristics were explored.

**Results:**

There is a significantly positive correlation between influenza transmissibility and five distinct ambient pollutants: PM_2.5_, PM_10_, SO_2_, CO, and NO_2_. On average, these ambient pollutants explained percentages of the variance in R_t_: 0.8%, 0.8%, 1.9%, 1.3%, and 1.4%, respectively. Conversely, O_3_ was found to be negatively associated with R_t_, explaining 1.5% of the variance in R_t_. When controlling for the effect of susceptibles depletion, the effects of all pollutants were more pronounced. The effects of PM_2.5_, PM_10_, CO, and SO_2_ were higher in the eastern and southern regions.

**Conclusions:**

Most ambient pollutants may potentially contribute to the facilitation of human‐to‐human influenza virus transmission in China. This observed association was maintained even after adjusting for variation in the susceptible population.

## 1 INTRODUCTION

Seasonal influenza is an acute respiratory infection caused by influenza viruses and has been a serious public health problem. It is estimated that there are approximately 1 billion cases of influenza worldwide each year, of which 3–5 million are severe, and 290,000–650,000 influenza‐associated respiratory deaths (case fatality rate 0.1%–0.2%).^1^, ^2^

Influenza can be transmitted by droplets made during infections with cough, sneeze, or talk. The amount of host‐virus excretion, population susceptibility, and viability of the influenza virus in the environment are three key factors that affect influenza transmissibility. Several studies have reported that human mobility,^3^ climate,^4^, ^5^ non‐pharmaceutical interventions,^6^ ambient pollutants,^7^ and the types of the virus^8^ affected the host‐to‐host transmission of influenza by acting on one or more of the above aspects.

Ambient air pollutants were considered potential drivers of influenza activity.^9^, ^10^, ^11^ For instance, Wang et al. demonstrated that particulate matter ≤2.5 μm (PM_2.5_) was significantly associated with an increased risk of pediatric seasonal influenza cases in Shijiazhuang.^10^ Yang and colleagues found that most air pollutants (including PM_2.5_, particulate matter ≤10 μm [PM_10_], nitrogen dioxide [NO_2_], sulfur dioxide [SO_2_], and carbon monoxide [CO]) were associated with an increased risk of influenza‐like illness (ILI) in 30 provinces in China.^9^ PM_10_ and ozone (O_3_) were associated with more pediatric influenza cases in Brisbane.^7^ However, little is known about the impact of exposure to ambient air pollutants on influenza transmissibility.

The dependent variable of most studies on the association between ambient pollutants and influenza is the absolute count of ILI.^11^, ^12^ Because of the differences in the distribution of sentinel hospitals and population size and density within cities, the number of ILI cases is not an ideal proxy for evaluating influenza transmissibility.^13^ Minor changes in influenza transmissibility could have a substantial effect on its incidence by dynamical resonance.^14^ For this reason, the daily effective instantaneous reproduction number (R_t_), defined as the average number of secondary infections caused by a typical single infectious individual at time t, is considered a more rational proxy for exploring the impact of environmental factors on influenza transmissibility than absolute ILI cases.^13^, ^15^

In addition, in previous studies, researchers considered the modification or confounding effects of environmental factors, such as temperature, humidity, and population size, on the association between ambient pollutants and influenza.^11^, ^12^, ^15^ But they did not pay enough attention to the impact of the depletion of susceptibles in the population with the development of the epidemic. The proportion of susceptible individuals in a population is a key determinant of the spread and size of an infectious disease epidemic^16^ and thus may directly or indirectly affect the association between environmental factors and influenza.

Therefore, the goals of our study were to examine the associations of six ambient pollutants (O_3_, PM_2.5_, PM_10_, NO_2_, SO_2_, and CO) with influenza transmissibility (R_t_) in 30 provinces of China and assess the effect of the depletion of susceptibles.

## 2 METHODS

### 2.1 Data source: 6 years of data from 2014 to 2019

Daily concentrations of six ambient pollutants in 30 provinces were obtained from the China High Air Pollutants (CHAP) dataset.^17^ Hourly ambient temperature and dew point temperature data were obtained from the China Meteorological Administration to calculate relative humidity and absolute humidity using the R package “humidity” (R software, version 4.2.1). Weekly ILI and viral detection rate data were obtained from the Chinese National Influenza Surveillance Network. Based on previous studies,^18^, ^19^ proxy measures of the weekly incidence rate were obtained by multiplying the ILI percentage among patients visiting sentinel hospitals with the proportion of influenza‐positive specimens. This proxy is considered a precise representation of the activity of an influenza infection.^20^, ^21^ We multiplied the weekly incidence rate by a constant (10,000), representing the inverse of the coverage of the sentinel sites in the studied provinces, and rounded the resulting values to the nearest integers to obtain a time series of weekly incidence rate counts.^22^

Influenza epidemics were defined as outbreaks exceeding the epidemic threshold for at least seven consecutive weeks or more. The epidemic threshold was determined as the 50th percentile of all the non‐zero weekly incidence rate counts over the study period.^21^, ^22^ Spline functions were used to interpolate the weekly incidence rate counts to produce daily incidence rate counts, which were used to estimate transmissibility.^21^, ^22^

### 2.2 R_t_ and adjusted R_t_ estimation

Daily R_t_, a real‐time measure of transmissibility, is estimated according to the Bayesian framework applied to the branching process model proposed by Cori et al.,^23^ which is an extension of the Fraser method.^24^ We assumed a gamma distribution with a mean of 2.6 days and a standard deviation of 1.5 days as the serial interval distribution.^25^ As an epidemic progresses, the number of susceptible individuals in the population will decrease, and R_t_ can gradually decrease as a result. Therefore, to adjust for this effect, we calculated the adjusted R_t_ according to Ali et al.^22^ The detailed estimation process of R_t_ and adjusted R_t_ can be found in Supporting information.

### 2.3 Statistical analyses

Our current statistical analysis strategy is composed of two parts: univariate analysis and multivariate analysis. First, we employed a log‐linear regression with a 0–7 day lag to identify significant environmental drivers of R_t_/adjusted R_t_. To ascertain whether the association between each environmental variable and R_t_/adjusted R_t_ occurred by chance, we performed a permutation test on these log regression models. This was done using 1000 dummy or null scenarios, and the results were compared with a real‐time series. Only when the *p*‐value from the permutation test was less than 0.05 was the corresponding environmental variable considered a significant driver of R_t_/adjusted R_t_. It was subsequently included in the multivariate analysis.

Secondly, we used distributed lag non‐linear models with a lag of 0–7 days (DLNM, R software, version 2.4.7) to quantify the impact of individual drivers. This was achieved by comparing R‐square (R^2^) values for Model 1 and Model 2. Model 1 evaluated the impacts of depletion in susceptibility over time and/or inter‐epidemic effects, temperature, and absolute humidity on Rt/adjusted Rt. Model 2 incorporated the additional effect of the respective ambient pollutant.

**FIGURE 1:**
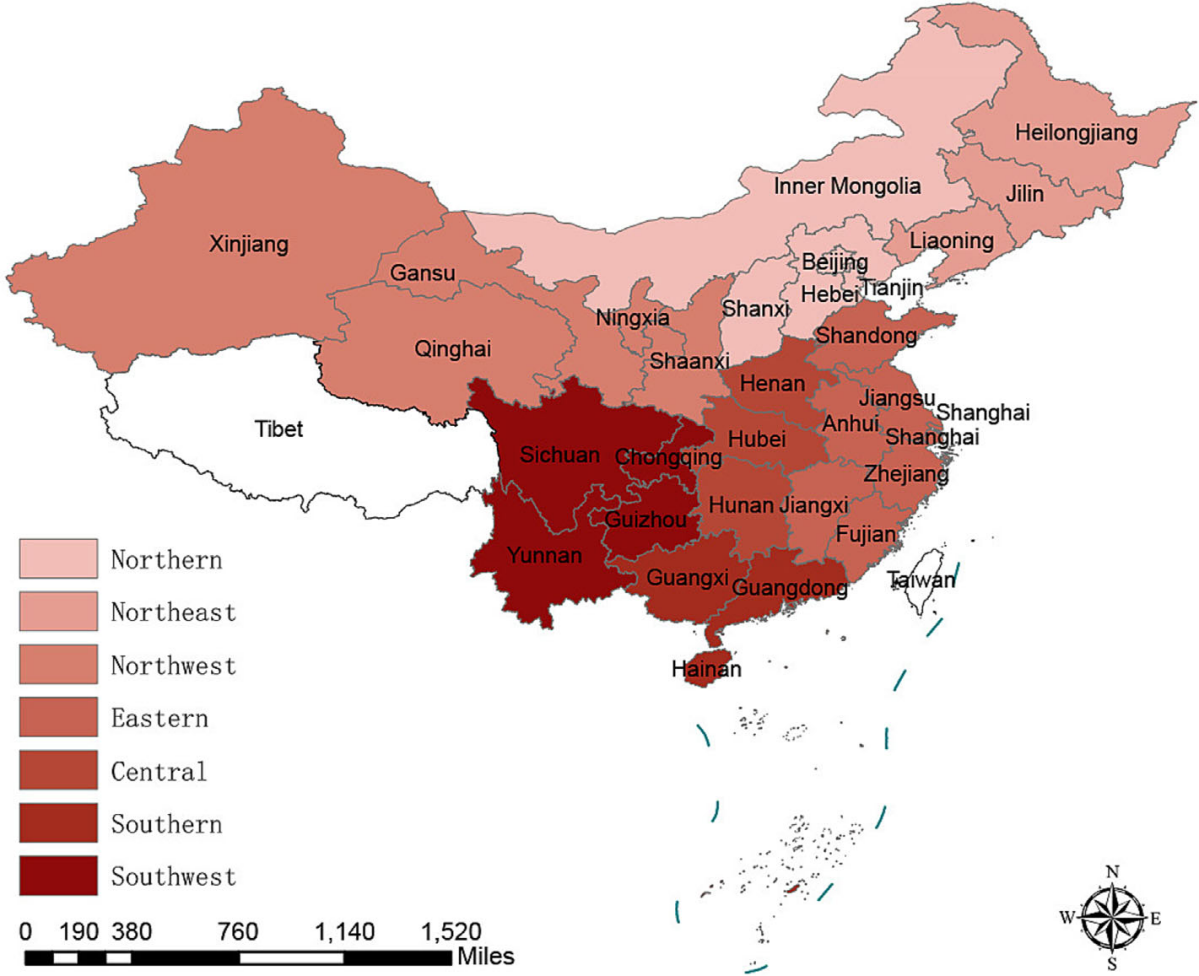
The 30 Chinese provinces were divided into seven regions. The Tibet autonomous region was excluded from our analysis due to its high proportion of missing data on influenza‐like illness.

To examine whether the impacts of ambient pollutants varied by region, we classified the 30 provinces into seven different regions (Figure 1), following the categorization used in a previous study.^9^ We then estimated the region‐specific associations between ambient pollutants and R_t_ by fitting models for each category.

## 3 RESULTS

### 3.1 Background characteristics by provinces

The median of the maximum R_t_ for all epidemics across the 30 provinces is 1.57, ranging from 1.36 to 2.04. Table 1 presents summary statistics of ambient pollutants, air temperature, and relative and absolute humidity in 30 Chinese provinces from 2014 to 2019. Generally, the O_3_ concentration was higher in the eastern and southern regions, while the concentrations of PM_2.5_, PM_10_, NO_2_, SO_2_, and CO were higher in the northern and northeast regions. The weather in the high‐latitude regions, such as the northern, northeast, and northwest areas, tended to be colder and drier.

**TABLE 1:** Summary of the instantaneous reproductive number (R_t_), ambient pollutants, and meteorological variables, presented as median and interquartile range (IQR), across 30 Chinese provinces from 2014 to 2019.

Province	O_3_ ($\mathrm{ug}/m^{3}$)	PM_2.5_ ($\mathrm{ug}/m^{3}$)	PM_10_ ($\mathrm{ug}/m^{3}$)	SO_2_ ($\mathrm{ug}/m^{3}$)	CO ($\mathrm{mg}/m^{3}$)	NO_2_ ($\mathrm{ug}/m^{3}$)	Temperature (°C)	Relative humidity (%)	Absolute humidity (g/m^3^)
Northern									
Beijing	0.9 (0.5, 1.5)	46.5 (22.2, 93.2)	77.9 (44.8, 130.7)	44.7 (30.1, 64.5)	43.6 (25.1, 60.6)	8.8 (4.3, 17.2)	14.4 (3.0, 22.3)	54.1 (38.0, 70.8)	6.4 (2.3, 12.7)
Tianjin	1.3 (0.9, 1.8)	60.3 (31.6, 101.5)	97.6 (64.8, 154.8)	53.9 (38.6, 71.7)	30.6 (18.2, 48.5)	20.1 (12.6, 36.1)	1.5 (−1.0, 7.0)	38.4 (28.5, 53.8)	2.3 (1.5, 3.6)
Hebei	1.5 (1.0, 2.1)	72.7 (43.5, 113.9)	129.1 (88.2, 186.3)	50.5 (38.8, 64.9)	37.5 (23.0, 53.6)	36.3 (22.0, 55.9)	−0.5 (−3.5, 5.1)	38.1 (29.0, 50.8)	2.0 (1.4, 2.9)
Shanxi	1.8 (1.4, 2.4)	66.1 (44.6, 102.0)	122.4 (92.1, 161.9)	42.2 (32.2, 52.4)	41.2 (26.9, 57.0)	69.0 (38.5, 114.3)	−1.8 (−4.5, 3.7)	38.6 (31.1, 50.0)	1.7 (1.3, 2.6)
Inner Mongolia	1.0 (0.7, 1.4)	38.5 (25.6, 56.4)	84.3 (57.3, 115.2)	32.7 (24.4, 42.7)	49.7 (35.3, 65.0)	28.5 (19.1, 41.6)	−17.2 (−22.5, −5.1)	63.4 (48.6, 68.9)	0.9 (0.6, 1.6)
Northeast									
Liaoning	1.2 (0.9, 1.6)	52.1 (35.2, 73.7)	90.5 (68.6, 118.3)	36.6 (27.9, 45.9)	45.0 (32.0, 61.4)	40.0 (27.1, 61.0)	−2.8 (−6.9, 3.6)	48.7 (41.2, 57.7)	2.1 (1.4, 3.0)
Jilin	1.1 (0.9, 1.3)	53.4 (36.3, 75.1)	84.5 (63.2, 113.1)	33.3 (25.6, 41.8)	48.7 (35.1, 63.6)	30.0 (19.1, 46.9)	−9.3 (−13.9, 0.2)	54.9 (41.5, 66.9)	1.4 (1.0, 2.0)
Heilongjiang	0.8 (0.7, 1.0)	43.1 (29.2, 61.7)	65.7 (47.8, 94.6)	27.4 (21.2, 35.4)	51.5 (37.8, 63.7)	22.5 (16.4, 32.5)	−14.5 (−18.5, −3.6)	60.2 (51.6, 68.3)	1.1 (0.8, 2.0)
Northwest									
Shaanxi	1.5 (1.2, 1.9)	70.0 (46.5, 107.2)	126.4 (95.7, 168.3)	48.7 (38.6, 59.0)	35.7 (22.9, 50.2)	24.6 (15.7, 34.4)	−1.4 (−5.6, 4.1)	34.4 (24.6, 45.2)	1.4 (1.0, 2.3)
Gansu	1.1 (0.8, 1.3)	46.1 (33.9, 59.1)	102.9 (80.8, 130.7)	33.4 (25.8, 42.2)	52.1 (42.3, 65.4)	30.3 (18.8, 45.7)	0.3 (−2.8, 5.5)	42.7 (33.1, 56.2)	2.0 (1.5, 3.2)
Qinghai	1.8 (1.2, 2.4)	55.5 (39.5, 73.1)	111.4 (84.4, 139.2)	42.9 (32.8, 54.5)	47.1 (33.6, 64.8)	25.3 (17.1, 33.5)	−7.4 (−9.6, −2.6)	33.8 (27.3, 43.2)	1.1 (0.8, 1.4)
Ningxia	1.1 (0.8, 1.4)	49.0 (34.7, 68.3)	104.8 (81.0, 135.3)	35.6 (26.3, 43.7)	44.8 (33.5, 59.0)	54.3 (31.8, 85.5)	−2.8 (−6.1, 1.2)	40.4 (32.5, 49.5)	1.6 (1.3, 2.1)
Xinjiang	1.6 (1.1, 2.2)	71.4 (35.9, 121.6)	115.1 (72.0, 177.2)	43.9 (33.4, 58.1)	31.2 (24.0, 45.2)	11.3 (8.2, 16.7)	−5.6 (−9.6, 2.9)	57.4 (41.4, 65.7)	1.9 (1.5, 2.6)
Eastern									
Shanghai	0.8 (0.6, 1.0)	40.3 (24.5, 62.7)	56.2 (38.9, 80.6)	43.0 (31.1, 58.8)	64.8 (43.9, 84.3)	11.9 (8.5, 16.9)	11.3 (6.8, 22.7)	70.8 (60.0, 81.0)	7.5 (5.1, 15.8)
Jiangsu	0.9 (0.8, 1.1)	52.2 (34.6, 74.1)	86.2 (60.2, 120.2)	37.4 (29.0, 50.0)	57.8 (40.6, 75.1)	16.7 (11.5, 23.8)	9.4 (4.7, 18.8)	66.8 (50.9, 78.6)	5.7 (3.8, 9.4)
Zhejiang	0.8 (0.7, 0.9)	38.1 (27.4, 55.3)	60.5 (44.1, 87.2)	34.7 (25.9, 46.4)	54.9 (39.7, 71.7)	11.1 (8.2, 15.4)	13.2 (8.4, 22.6)	76.2 (65.6, 84.8)	8.7 (5.9, 16.1)
Anhui	0.9 (0.7, 1.1)	54.1 (38.9, 78.0)	83.3 (60.7, 112.4)	34.4 (26.1, 45.5)	48.5 (38.7, 63.0)	17.0 (12.5, 22.4)	9.3 (4.6, 20.0)	76.6 (59.1, 86.0)	6.3 (4.4, 12.4)
Fujian	0.7 (0.6, 0.9)	25.0 (17.5, 34.4)	44.6 (33.5, 60.4)	23.0 (18.2, 28.7)	50.0 (39.1, 62.3)	8.5 (6.9, 10.9)	20.2 (16.0, 27.7)	76.8 (68.8, 84.4)	14.1 (10.1, 21.7)
Jiangxi	1.0 (0.8, 1.1)	37.9 (28.3, 56.3)	62.4 (46.0, 90.6)	23.8 (17.6, 32.0)	47.7 (34.9, 60.2)	19.8 (13.9, 27.6)	16.6 (9.7, 24.8)	78.8 (67.8, 86.5)	10.9 (7.1, 18.7)
Shandong	1.2 (0.9, 1.6)	70.2 (43.6, 106.5)	127.4 (93.2, 174.4)	44.5 (34.4, 56.2)	45.5 (30.0, 64.5)	32.7 (20.6, 47.4)	2.5 (−0.3, 7.1)	51.0 (41.6, 62.0)	3.1 (2.2, 4.2)
Central									
Henan	1.4 (1.1, 1.8)	78.4 (49.4, 120.1)	132.2 (97.6, 187.0)	46.6 (33.8, 57.8)	41.4 (26.9, 61.1)	26.6 (15.1, 44.3)	6.7 (1.8, 13.5)	46.7 (31.9, 66.8)	3.4 (2.2, 5.8)
Hubei	1.1 (1.0, 1.4)	54.8 (37.7, 82.6)	87.9 (64.3, 120.9)	34.3 (25.2, 44.2)	51.2 (35.4, 65.8)	13.7 (9.9, 19.9)	12.3 (6.8, 21.1)	72.1 (60.9, 82.4)	7.5 (5.1, 13.4)
Hunan	1.0 (0.9, 1.2)	43.7 (29.2, 63.0)	68.5 (47.2, 98.3)	24.7 (18.3, 33.5)	51.9 (38.7, 64.3)	15.8 (9.9, 22.5)	15.8 (8.5, 23.6)	76.8 (61.8, 86.6)	8.9 (5.4, 17.0)
Southern									
Guangdong	0.8 (0.7, 0.9)	26.8 (18.9, 38.3)	43.6 (33.4, 60.2)	25.6 (20.6, 33.3)	49.1 (37.9, 65.5)	10.0 (8.2, 12.4)	23.1 (17.7, 27.2)	79.3 (71.6, 85.2)	16.9 (10.7, 22.4)
Guangxi	0.9 (0.8, 1.0)	28.6 (20.1, 40.4)	47.5 (35.3, 63.5)	19.3 (16.2, 24.6)	49.3 (39.2, 60.9)	11.7 (9.9, 15.2)	21.0 (13.3, 25.8)	81.1 (72.1, 88.7)	14.8 (8.6, 20.6)
Hainan	0.6 (0.5, 0.7)	15.8 (11.7, 21.7)	31.5 (24.6, 39.0)	12.0 (9.9, 14.7)	51.4 (41.0, 67.7)	4.2 (3.8, 5.0)	25.0 (21.2, 28.0)	79.9 (73.7, 85.6)	18.6 (15.5, 22.0)
Southwest									
Chongqing	0.9 (0.8, 1.1)	41.2 (28.5, 61.1)	67.6 (46.6, 90.0)	41.7 (34.1, 51.4)	29.4 (17.0, 51.6)	10.7 (8.0, 14.0)	13.8 (8.5, 19.8)	75.8 (67.0, 83.5)	8.7 (6.5, 12.7)
Sichuan	0.9 (0.7, 1.1)	44.2 (30.3, 67.2)	71.9 (51.8, 102.8)	32.2 (26.4, 39.6)	44.3 (32.2, 58.8)	14.9 (11.9, 18.8)	8.0 (2.7, 14.3)	49.5 (39.5, 65.3)	3.8 (2.3, 8.0)
Guizhou	0.7 (0.6, 0.9)	33.3 (23.4, 46.9)	55.1 (38.4, 75.6)	24.4 (19.4, 30.0)	48.2 (35.6, 60.9)	14.5 (9.7, 23.7)	10.4 (4.7, 15.9)	78.8 (63.7, 88.4)	6.3 (4.7, 10.3)
Yunnan	0.9 (0.8, 1.0)	27.2 (20.2, 34.6)	50.5 (39.6, 62.2)	20.9 (17.7, 24.0)	51.8 (40.7, 73.7)	13.2 (10.9, 16.7)	14.2 (11.3, 18.3)	62.5 (52.9, 71.2)	7.4 (6.1, 9.3)

Abbreviations: CO, carbon monoxide; NO_2_, nitrogen dioxide; O_3_, ozone; PM_10_, particulate matter ≤10 μm; PM_2.5_, particulate matter ≤2.5 μm; SO_2_, sulfur dioxide.

### 3.2 Univariate regression model

We explored the association between influenza transmissibility, as measured by R_t_/adjusted R_t_ and each factor with lagged values of 0–7 days for each province. Except for O_3_, other pollutants (including PM_2.5_, PM_10_, SO_2_, NO_2_, and CO) have a positive correlation with R_t_/adjusted R_t_ (data did not show). Tables S1 and S2 show the results from the non‐linear regression model and permutation test. Air temperature, relative humidity, absolute humidity, and six ambient pollutants were significantly correlated with R_t_/adjusted R_t_ in almost all provinces, although the variance of the R_t_/adjusted R_t_ explained by each driver was marginal.

Permutation analysis indicated that a substantially lesser variance in transmissibility was accounted for by the 1000 null/dummy time series for each respective driver when compared with the observed true time series. The difference in variance between the permutation analysis and the observed true time series was found to be statistically significant in most locations. These significant drivers were included in further analysis.

### 3.3 Multivariable regression analysis

In stratified multivariate regression analysis of R_t_ by location, Model 2—which incorporates all influencers such as depletion of susceptibles and/or inter‐epidemic factors and environmental factors—explained 44.2% of the observed variance in the R_t_, with a range of 20.8% to 66.9%. Among these, Model 1 (Table 2) explained a significant proportion of the observed variance in R_t_ (between 12.1% and 54.4%) by incorporating factors such as depletion of susceptibles and/or inter‐epidemic factors and meteorological considerations (air temperature and absolute humidity).

**TABLE 2:** Percentage of the variance of the instantaneous reproduction number (R_t_) explained by the ambient pollutants, from models on pre‐defined influenza epidemics in respective locations from 2014 to 2019. The results based on the distributed lag model (DLNM) with lags of 0–7 days.

Province	Models	With unadjusted R_t_ $\left(R^{2}\text{or}\;R^{2}\;(\%∆R^{2})\right)$								With adjusted R_t_ $\left(R^{2}\text{or}\;R^{2}\;(\%∆R^{2})\right)$							
		$R^{2}$	O_3_	PM_2.5_	PM_10_	SO_2_	CO	NO_2_	All	$R^{2}$	O_3_	PM_2.5_	PM_10_	SO_2_	CO	NO_2_	All
Northern																	
Beijing	Model 1^a^	0.35	‐	‐	‐	‐	‐	‐	‐	0.19	‐	‐	‐	‐	‐	‐	‐
	Model 2^b^	‐	0.37 (2.9)	0.35 (0.9)	1.16 (0.4)	0.36 (1.3)	0.35 (0.6)	0.37 (2.0)	0.46 (11.9)	‐	0.21 (1.3)	0.20 (1.1)	1.32 (0.2)	0.23 (3.5)	0.20 (0.8)	0.21 (1.9)	0.37 (17.4)
Tianjin	Model 1^b^	0.45	‐	‐	‐	‐	‐	‐	‐	0.21	‐	‐	‐	‐	‐	‐	‐
	Model 2^b^	‐	0.46 (1.5)	0.46 (0.7)	1.08 (0.5)	0.47 (1.8)	0.47 (2.3)	0.45 (0.1)	0.54 (8.7)	‐	0.41 (20.1)	0.23 (1.5)	2.29 (0.2)	0.24 (2.5)	0.23 (1.8)	0.28 (6.8)	0.45 (23.5)
Hebei	Model 1^a^	0.48	‐	‐	‐	‐	‐	‐	‐	0.29	‐	‐	‐	‐	‐	‐	‐
	Model 2^b^	‐	0.49 (0.8)	0.49 (0.3)	0.24 (0.5)	0.51 (2.7)	0.50 (1.8)	0.48 (0.1)	0.59 (11.1)	‐	0.47 (17.1)	0.32 (2.6)	3.36 (0.3)	0.32 (2.9)	0.33 (3.8)	0.41 (11.2)	0.55 (25.4)
Shanxi	Model 1^a^	0.30	‐	‐	‐	‐	‐	‐	‐	0.24	‐	‐	‐	‐	‐	‐	‐
	Model 2^b^	‐	0.39 (8.8)	0.33 (3.1)	2.94 (0.3)	0.34 (4.6)	0.33 (3.3)	0.37 (7.0)	0.52 (21.9)	‐	0.38 (13.6)	0.29 (4.5)	3.82 (0.3)	0.30 (5.6)	0.30 (5.2)	0.29 (5.1)	0.50 (25.2)
Inner Mongolia	Model 1^a^	0.48	‐	‐	‐	‐	‐	‐	‐	0.19	‐	‐	‐	‐	‐	‐	‐
	Model 2^b^	‐	0.40 (0.3)	0.43 (2.7)	1.90 (0.4)	0.45 (4.9)	0.41 (1.0)	0.41 (0.7)	0.50 (10.0)	‐	0.48 (29.1)	0.25 (6.4)	1.28 (0.2)	0.25 (6.8)	0.31 (12.2)	0.31 (12.4)	0.50 (31.3)
Northeast																	
Liaoning	Model 1^a^	0.40	‐	‐	‐	‐	‐	‐	‐	0.31	‐	‐	‐	‐	‐	‐	‐
	Model 2^b^	‐	0.41 (1.8)	0.40 (0.1)	0.60 (0.4)	0.40 (0.3)	0.40 (0.3)	0.40 (0.1)	0.45 (5.4)	‐	0.42 (11.0)	0.32 (1.0)	0.95 (0.3)	0.34 (3.1)	0.33 (2.2)	0.33 (2.4)	0.46 (15.6)
Jilin	Model 1^a^	0.44	‐	‐	‐	‐	‐	‐	‐	0.2	‐	‐	‐	‐	‐	‐	‐
	Model 2^b^	‐	0.45 (0.2)	0.45 (1.1)	1.58 (0.5)	0.45 (0.9)	0.45 (0.2)	0.45 (0.2)	0.51 (6.4)	‐	0.37 (15.9)	0.24 (2.1)	2.92 (0.2)	0.25 (3.8)	0.23 (1.4)	0.24 (2.0)	0.49 (27.7)
Heilongjiang	Model 1^a^	0.31	‐	‐	‐	‐	‐	‐	‐	0.28	‐	‐	‐	‐	‐	‐	‐
	Model 2^b^	‐	0.32 (0.7)	0.32 (0.4)	0.15 (0.3)	0.35 (3.7)	0.31 (0.1)	0.32 (1.0)	0.43 (11.8)	‐	0.43 (15.2)	0.29 (1.6)	1.55 (0.3)	0.37 (9.3)	0.40 (12.1)	0.40 (12.3)	0.55 (27.7)
Northwest																	
Shaanxi	Model 1^a^	0.42	‐	‐	‐	‐	‐	‐	‐	0.34	‐	‐	‐	‐	‐	‐	‐
	Model 2^b^	‐	0.43 (1.4)	0.42 (0.1)	0.21 (0.4)	0.42 (0.8)	0.42 (0.6)	0.44 (2.5)	0.51 (9.5)	‐	0.60 (26.4)	0.35 (1.0)	0.84 (0.3)	0.36 (2.3)	0.36 (2.4)	0.42 (8.0)	0.63 (29.0)
Gansu	Model 1^a^	0.40	‐	‐	‐	‐	‐	‐	‐	0.30	‐	‐	‐	‐	‐	‐	‐
	Model 2^b^	‐	0.43 (2.5)	0.40 (0.2)	0.00 (0.4)	0.44 (4.2)	0.43 (3.3)	0.40 (0.4)	0.50 (9.7)	‐	0.48 (18.2)	0.32 (2.2)	0.91 (0.3)	0.35 (4.7)	0.35 (4.8)	0.40 (9.5)	0.53 (22.6)
Qinghai	Model 1^a^	0.39	‐	‐	‐	‐	‐	‐	‐	0.36	‐	‐	‐	‐	‐	‐	‐
	Model 2^b^	‐	0.40 (1.2)	0.40 (0.9)	1.63 (0.4)	0.42 (3.4)	0.40 (1.5)	0.42 (2.7)	0.49 (10.6)	‐	0.58 (22.5)	0.42 (6.3)	2.40 (0.4)	0.47 (11.2)	0.48 (12.0)	0.62 (26.5)	0.68 (32.3)
Ningxia	Model 1^a^	0.37	‐	‐	‐	‐	‐	‐	‐	0.10	‐	‐	‐	‐	‐	‐	‐
	Model 2^b^	‐	0.37 (0.0)	0.40 (2.3)	2.62 (0.4)	0.38 (0.1)	0.39 (1.2)	0.38 (0.2)	0.45 (7.9)	‐	0.15 (4.6)	0.15 (4.3)	7.20 (0.2)	0.11 (0.9)	0.11 (0.4)	0.11 (1.1)	0.31 (20.9)
Xinjiang	Model 1^a^	0.49	‐	‐	‐	‐	‐	‐	‐	0.43	‐	‐	‐	‐	‐	‐	‐
	Model 2^b^	‐	0.51 (2.0)	0.49 (0.1)	0.02 (0.5)	0.50 (1.7)	0.50 (1.4)	0.50 (0.8)	0.55 (6.6)	‐	0.48 (4.8)	0.47 (3.6)	1.46 (0.4)	0.44 (0.4)	0.44 (1.2)	0.46 (3.2)	0.55 (11.9)
Eastern																	
Shanghai	Model 1^a^	0.51	‐	‐	‐	‐	‐	‐	‐	0.07	‐	‐	‐	‐	‐	‐	‐
	Model 2^b^	‐	0.52 (1.6)	0.51 (0.6)	1.20 (0.5)	0.51 (0.4)	0.51 (0.2)	0.52 (1.6)	0.57 (6.2)	‐	0.22 (14.5)	0.10 (2.8)	3.98 (0.1)	0.12 (4.4)	0.12 (4.6)	0.23 (16.0)	0.31 (24.1)
Jiangsu	Model 1^a^	0.39	‐	‐	‐	‐	‐	‐	‐	0.21	‐	‐	‐	‐	‐	‐	‐
	Model 2^b^	‐	0.42 (3.0)	0.40 (0.9)	0.41 (0.4)	0.42 (3.0)	0.40 (1.5)	0.44 (5.5)	0.56 (17.3)	‐	0.33 (11.1)	0.24 (3.0)	4.03 (0.3)	0.30 (8.5)	0.28 (7.0)	0.26 (4.2)	0.44 (22.7)
Zhejiang	Model 1^a^	0.34	‐	‐	‐	‐	‐	‐	‐	0.06	‐	‐	‐	‐	‐	‐	‐
	Model 2^b^	‐	0.35 (0.6)	0.35 (0.4)	0.61 (0.4)	0.35 (0.6)	0.35 (0.1)	0.36 (2.0)	0.45 (10.6)	‐	0.27 (21.0)	0.16 (9.8)	9.79 (0.2)	0.17 (11.3)	0.19 (13.4)	0.21 (14.9)	0.37 (30.5)
Anhui	Model 1^a^	0.44	‐	‐	‐	‐	‐	‐	‐	0.22	‐	‐	‐	‐	‐	‐	‐
	Model 2^b^	‐	0.44 (0.1)	0.45 (0.7)	0.61 (0.4)	0.45 (1.3)	0.44 (0.1)	0.45 (1.3)	0.49 (4.7)	‐	0.37 (14.8)	0.25 (3.0)	3.58 (0.3)	0.33 (10.6)	0.27 (4.8)	0.28 (5.3)	0.47 (24.9)
Fujian	Model 1^a^	0.24	‐	‐	‐	‐	‐	‐	‐	0.06	‐	‐	‐	‐	‐	‐	‐
	Model 2^b^	‐	0.25 (1.1)	0.24 (0.7)	0.31 (0.2)	0.26 (2.4)	0.28 (3.8)	0.24 (0.1)	0.37 (13.4)	‐	0.23 (17.1)	0.08 (1.2)	5.85 (0.1)	0.23 (17.1)	0.14 (8.1)	0.18 (11.4)	0.44 (37.8)
Jiangxi	Model 1^a^	0.31	‐	‐	‐	‐	‐	‐	‐	0.04	‐	‐	‐	‐	‐	‐	‐
	Model 2^b^	‐	0.31 (0.2)	0.31 (0.2)	0.11 (0.3)	0.31 (0.2)	0.31 (0.4)	0.32 (1.2)	0.35 (4.9)	‐	0.06 (2.0)	0.11 (6.7)	6.60 (0.1)	0.19 (15.1)	0.13 (8.6)	0.13 (9.0)	0.23 (19.1)
Shandong	Model 1^a^	0.54	‐	‐	‐	‐	‐	‐	‐	0.22	‐	‐	‐	‐	‐	‐	‐
	Model 2^b^	‐	0.58 (3.7)	0.55 (0.4)	0.55 (0.5)	0.59 (4.8)	0.56 (1.5)	0.56 (2.0)	0.67 (12.4)	‐	0.52 (30.5)	0.27 (4.9)	6.69 (0.3)	0.40 (18.5)	0.34 (11.9)	0.38 (16.1)	0.59 (37.4)
Central																	
Henan	Model 1^a^	0.37	‐	‐	‐	‐	‐	‐	‐	0.22							
	Model 2^b^	‐	0.38 (1.2)	0.37 (0.4)	0.47 (0.4)	0.38 (1.2)	0.38 (1.1)	0.38 (1.2)	0.47 (10.1)	‐	0.48 (26.4)	0.23 (1.3)	2.74 (0.2)	0.28 (6.0)	0.27 (5.5)	0.41 (19.7)	0.53 (31.6)
Hubei	Model 1^a^	0.32	‐	‐	‐	‐	‐	‐	‐	0.14	‐	‐	‐	‐	‐	‐	‐
	Model 2^b^	‐	0.32 (0.9)	0.32 (0.0)	0.00 (0.3)	0.32 (0.6)	0.32 (0.0)	0.32 (0.3)	0.34 (2.6)	‐	0.23 (9.0)	0.20 (6.1)	5.69 (0.2)	0.21 (7.8)	0.21 (6.9)	0.19 (5.4)	0.30 (16.2)
Hunan	Model 1^a^	0.20	‐	‐	‐	‐	‐	‐	‐	0.17	‐	‐	‐	‐	‐	‐	‐
	Model 2^b^	‐	0.20 (0.2)	0.21 (1.3)	0.66 (0.2)	0.21 (1.3)	0.20 (0.1)	0.21 (1.4)	0.27 (7.3)	‐	0.23 (5.9)	0.20 (3.2)	3.06 (0.2)	0.28 (10.8)	0.28 (11.0)	0.18 (0.9)	0.40 (23.3)
Southern																	
Guangdong	Model 1^a^	0.23	‐	‐	‐	‐	‐	‐	‐	0.04	‐	‐	‐	‐	‐	‐	‐
	Model 2^b^	‐	0.25 (2.2)	0.24 (1.3)	1.82 (0.2)	0.23 (0.9)	0.23 (0.4)	0.25 (2.5)	0.33 (9.9)	‐	0.05 (0.9)	0.14 (9.7)	12.17 (0.2)	0.34 (29.5)	0.18 (14.2)	0.15 (10.2)	0.40 (35.7)
Guangxi	Model 1^a^	0.24	‐	‐	‐	‐	‐	‐	‐	0.10	‐	‐	‐	‐	‐	‐	‐
	Model 2^b^	‐	0.26 (2.3)	0.24 (0.5)	0.53 (0.2)	0.25 (0.6)	0.25 (1.1)	0.25 (1.2)	0.30 (6.1)	‐	0.11 (0.5)	0.11 (0.8)	1.48 (0.1)	0.31 (21.0)	0.21 (11.4)	0.17 (7.2)	0.47 (36.4)
Hainan	Model 1^a^	0.20	‐	‐	‐	‐	‐	‐	‐	0.10	‐	‐	‐	‐	‐	‐	‐
	Model 2^b^	‐	0.20 (0.3)	0.20 (0.2)	0.41 (0.2)	0.20 (0.2)	0.20 (0.4)	0.20 (0.4)	0.24 (4.2)	‐	0.12 (2.6)	0.14 (3.8)	5.71 (0.2)	0.14 (4.1)	0.22 (12.0)	0.11 (1.4)	0.31 (20.8)
Southern																	
Chongqing	Model 1^a^	0.26	‐	‐	‐	‐	‐	‐	‐	0.11	‐	‐	‐	‐	‐	‐	‐
	Model 2^b^	‐	0.27 (1.0)	0.29 (3.0)	1.86 (0.3)	0.30 (4.5)	0.31 (5.0)	0.27 (1.1)	0.39 (12.7)	‐	0.32 (20.3)	0.15 (3.3)	3.20 (0.1)	0.28 (16.6)	0.15 (3.3)	0.22 (10.6)	0.48 (36.3)
Sichuan	Model 1^a^	0.31	‐	‐	‐	‐	‐	‐	‐	0.08	‐	‐	‐	‐	‐	‐	‐
	Model 2^b^	‐	0.32 (1.3)	0.32 (1.3)	0.91 (0.3)	0.33 (2.5)	0.32 (1.0)	0.31 (0.4)	0.40 (9.1)	‐	0.10 (2.7)	0.08 (0.6)	0.50 (0.1)	0.08 (0.2)	0.08 (0.1)	0.08 (0.3)	0.13 (5.0)
Guizhou	Model 1^a^	0.34	‐	‐	‐	‐	‐	‐	‐	0.21	‐	‐	‐	‐	‐	‐	‐
	Model 2^b^	‐	0.34 (0.0)	0.34 (0.0)	0.16 (0.3)	0.35 (0.4)	0.36 (1.4)	0.34 (0.0)	0.40 (5.4)	‐	0.25 (3.3)	0.23 (1.2)	0.89 (0.2)	0.25 (4.1)	0.22 (0.9)	0.22 (0.4)	0.34 (12.4)
Yunnan	Model 1^a^	0.12	‐	‐	‐	‐	‐	‐	‐	0.08	‐	‐	‐	‐	‐	‐	‐
	Model 2^b^	‐	0.13 (0.9)	0.12 (0.2)	0.32 (0.1)	0.13 (0.6)	0.14 (1.6)	0.14 (1.5)	0.21 (8.8)	‐	0.18 (10.1)	0.09 (0.8)	0.82 (0.1)	0.17 (9.5)	0.11 (3.0)	0.08 (0.6)	0.26 (18.8)

Abrreviations: CO, carbon monoxide; NO_2_, nitrogen dioxide; O_3_, ozone; PM_10_, particulate matter ≤10 μm; PM_2.5_, particulate matter ≤2.5 μm; R^2^, R‐square; SO_2_, sulfur dioxide.

^a^ Model 1: Factors affecting $R_{t}$ (or adjusted $R_{t}$) include depletion of susceptibles and/or inter‐epidemic factors and environmental factors (air temperature, absolute humidity).

^b^ Model 2: Model 1 plus the respective ambient pollutant drivers.

When each environmental pollutant was incorporated separately into the multivariate regression model, there was a slight improvement in the model fit (R^2^). Specifically, O_3_, PM_2.5_, PM_10_, SO_2_, CO, and NO_2_, respectively, accounted for an additional average variance (minimum, maximum) in R_t_ of 1.5% (0%, 8.8%), 0.8% (0%, 3.1%), 0.8% (0%, 2.9%), 1.9% (0.1%, 4.9%), 1.3% (0%, 5.0%), and 1.4% (0.0%, 7.0%).

In the adjusted R_t_ analysis, the inclusion of each environmental pollutant explained a larger variance in the R_t_ at the studied location compared with the R_t_. O_3_, PM_2.5_, PM_10_, SO_2_, CO, and NO_2_ accounted for an additional 13.1% (0.5%–30.5%), 3.3% (0.6%–9.8%), 3.7% (0.5%–12.2%), 8.4% (0.2%–29.5%), 6.2% (0.1%–14.2%), and 7.9% (0.3%–26.5%) of the variance, respectively. When the impacts of all pollutants were considered together, they explained 24.8% of the variance in the adjusted R_t_, with a range of 4.9% to 37.8%.

### 3.4 Estimates of the variance of R_t_/adjusted R_t_ variation across regions

**FIGURE 2:**
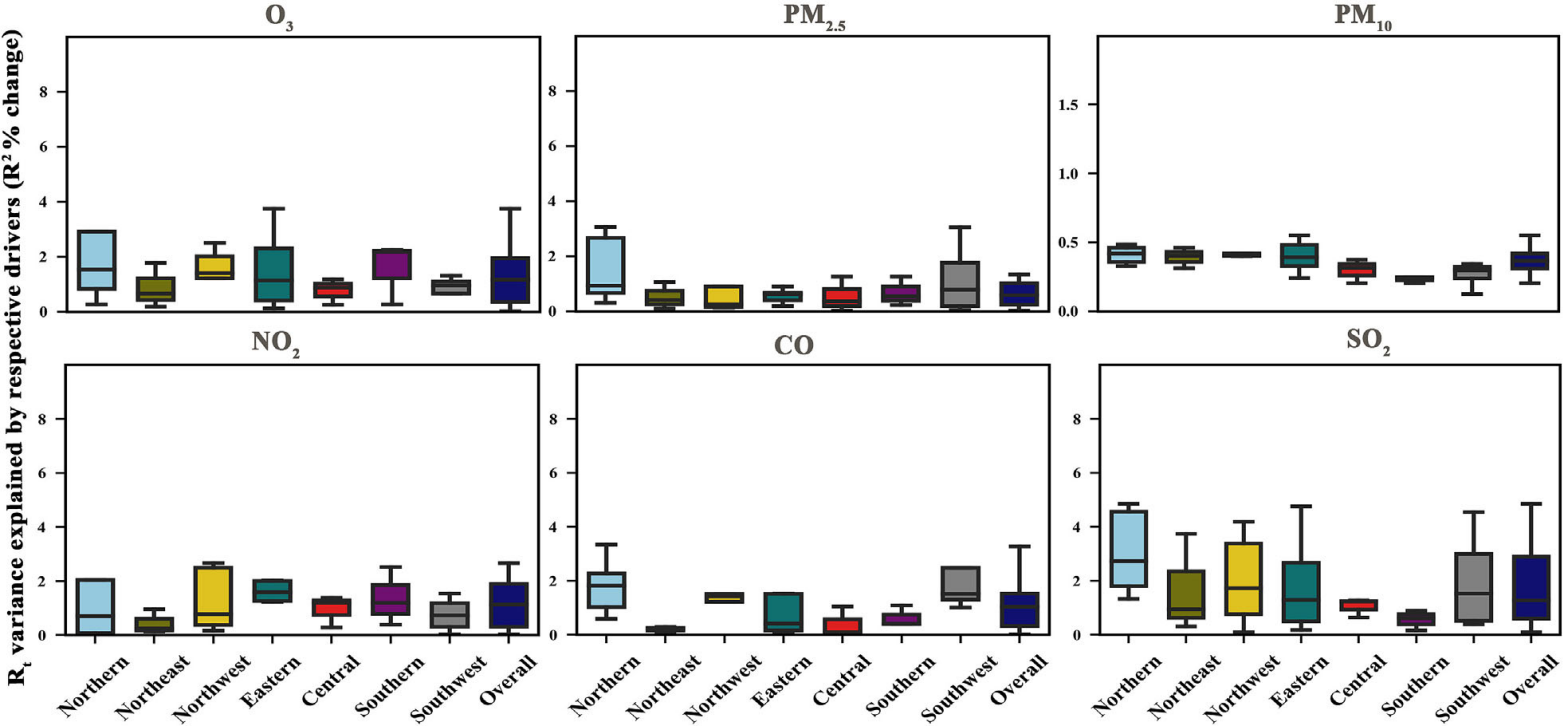
Estimated percentage changes of the variance of the instantaneous reproduction number (R_t_) explained by the respective drivers.

**FIGURE 3:**
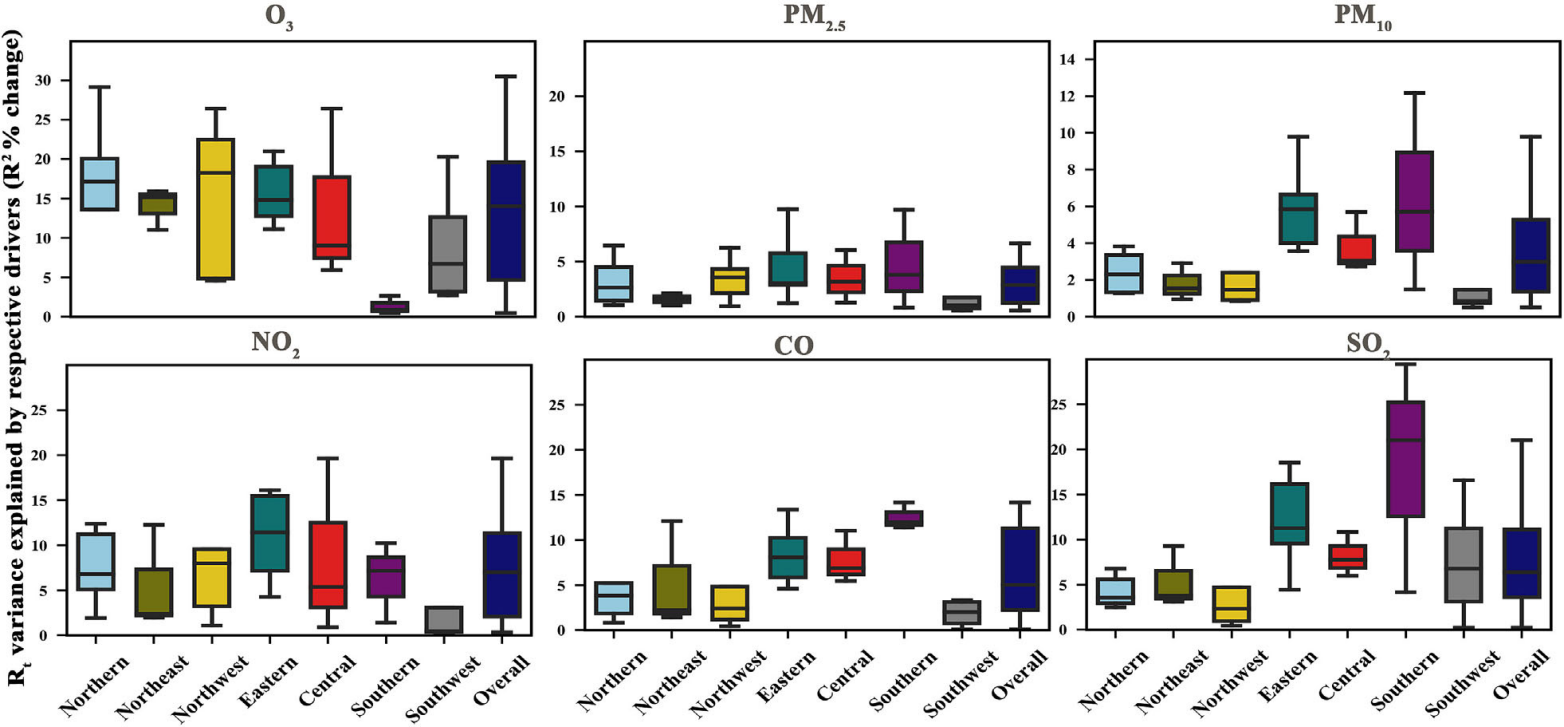
Estimated percentage changes of the variance of the adjusted instantaneous reproduction number (R_t_) explained by the respective drivers.

Figures 2 and 3 illustrate the regional variations in the impact of ambient pollutants on the R_t_/adjusted R_t_. Across all pollutants, the influence of ambient pollutants on Rt intensified after accounting for the effect of the depletion of susceptibles, as demonstrated in Figure 3. In scenarios where the effect of depletion of susceptibles was not considered, the impacts of pollutants on R_t_ did not exhibit substantial regional disparities, with the exception of CO. By factoring in the effect of the depletion of susceptibles, the proportion of R_t_ attributable to all pollutants increased.

In particular, the contribution of O_3_ to the adjusted R_t_ in the southern region was weaker than in other regions. The adjusted R_t_ in eastern and southern regions appeared more sensitive to the influence of PM (PM_2.5_, PM_10_), CO, and SO_2_. In contrast, the northern regions were more susceptible to the effects of O_3_ and NO_2_.

## 4 DISCUSSION

In this comprehensive nationwide study, we have evaluated influenza transmissibility across diverse locations in 30 provinces in China. Our results indicate that the R_t_ exhibits a significant positive correlation with PM_2.5_, PM_10_, SO_2_, NO_2_, and CO, while showing a negative correlation with O_3_. It is noteworthy that the change in depletion of susceptibles is one of the key factors influencing the transmissibility of influenza during epidemic outbreaks, while other extrinsic drivers may additionally contribute to changes in transmissibility. The associations between ambient pollutants and R_t_ displayed minor regional variations. The findings from our research may aid in understanding the profound influence of air pollution on influenza transmissibility, thereby helping to formulate effective prevention strategies aimed at decreasing the spread of this infectious disease.

Exposure to ambient pollutants can detrimentally impact lung function and exacerbate a range of diseases, such as asthma and cardiovascular disease. Through this research, we have discerned statistically significant positive correlations between ambient pollutant exposure and the increased transmissibility of influenza. Various population studies have indicated that heightened concentrations of ambient pollutants can lead to an increased susceptibility to chronic respiratory diseases, including but not limited to lung cancer, chronic obstructive pulmonary disease, and asthma.^26^ Moreover, a consistent body of epidemiological studies has emphatically demonstrated a strong correlation between air pollution and the increased risk of influenza.^7^, ^9^, ^11^ Our results, stemming from an analysis of five ambient pollutants and their relation to influenza transmissibility, align with prior studies' conclusions: exposure to most ambient pollutants is commonly linked to adverse health outcomes, whether they be chronic or infectious diseases. Interestingly, our study reveals a negative correlation between O3 and influenza transmissibility, a finding consistent with several prior studies.^9^, ^15^, ^27^ For example, Su and colleagues found a negative association between O_3_ and ILI in the city of Jinan, China,^27^ and Ali et al. reported reduced influenza transmissibility associated with O_3_ in Hong Kong, China.^15^

Population immunity is one of the key factors affecting the spread of influenza. With the development of the epidemic, the proportion of susceptibles decreases, population immunity gradually increases, and the transmissibility of influenza gradually decreases until the end of the epidemic. Previous research on the impact of environmental factors on influenza transmissibility has demonstrated that a significant portion of the variance in transmissibility is attributable to the depletion of susceptibles and humidity.^15^, ^22^ Therefore, it may be difficult to determine the impact of pollutants on influenza transmissibility without controlling for the effect of the depletion of susceptibles in the model. In the current study, we further analyzed the associations of ambient pollutants with adjusted R_t_. Our results showed that the associations between ambient pollutants and influenza transmissibility remained significant after considering the effect of the depletion of susceptibles. Concentrations of ambient pollutants have been associated with increased influenza transmissibility, except for O_3_. Based on these findings, our study suggests the necessity of acknowledging the role of ambient pollutants in exacerbating the spread of influenza. Therefore, when formulating prevention and control strategies, the implementation of measures aimed at reducing environmental pollution should be duly considered.

In the multivariable regression analysis, we found that a large proportion of the variance was explained by the intrinsic factors and climate factors in the basic model for influenza transmissibility; the ambient O_3_, PM_2.5_, PM_10_, SO_2_, CO, and NO_2_ contributed only marginally, average explaining a further 1.5%, 0.8%, 0.8%, 1.9%, 1.3%, and 1.4% of the variance, respectively. In evaluating the effect of depletion of susceptibles, we noticed an amplified proportion of variance in transmissibility attributable to ambient pollutants. This outcome underscores the significance of incorporating or appropriately controlling for factors that influence disease occurrence and progression in future research on environmental drivers of respiratory infectious diseases. Such an approach can more effectively shed light on the complex association between environmental factors and the incidence of infectious diseases.

The mechanisms underlying the association between air pollution and influenza transmissibility are less elucidated. One potential explanation for our findings might be that exposure to air pollution induces oxidative stress, impairs the activation of macrophage‐dependent invasive pathogens, and exacerbates inflammation.^28^ These factors can collectively harm the respiratory system, diminishing its resistance to viral and bacterial infections. Furthermore, ambient pollutants, specifically PM of smaller diameters that can remain suspended in the air for extended periods, have been posited as vectors for the transmission of various respiratory pathogens.^29^, ^30^ For example, PM particles with diameters less than 10 μm have been shown to facilitate the formation of condensation nuclei for influenza viruses, thereby increasing the spread of the virus.^31^ Evidence from a randomized, double‐blind trial suggests that NO_2_ may independently enhance the susceptibility of adults to respiratory virus infections.^32^ Animal studies indicate that sustained exposure to low levels of SO_2_ can intensify the severity of an influenza infection and magnify the deleterious effects of the virus.^33^ Moreover, we found a negative association between O_3_ and R_t_ in our study, which aligns with findings from several prior studies. This observed reduction in influenza transmissibility in relation to O_3_ might be attributable to the virucidal activity of O_3_ and its effect on host defense mechanism. In vitro studies have reported that O_3_ can inactivate the influenza virus within hours,^34^ and animal toxicology studies have shown a decrease in inflammation, injury, and oxidative stress following exposure to O_3_.^35^

China, as a vast country with diverse regions, might observe varying impacts of ambient pollutants on influenza transmissibility due to regional differences. Our study has discovered that the influence of ambient pollutants on transmissibility is stronger in the eastern and southern regions. Our findings align with a recent epidemiological study that reported that the effects of air pollutants on ILI in the eastern and central regions of China were higher than those in other regions.^9^ The exact underlying mechanisms of the heightened effects of ambient pollutants on transmissibility remain unclear and require further research. The stronger effect of ambient pollution in the eastern and southern regions may be partly attributable to personal exposure patterns. For instance, these regions generally offer more pleasant climates, leading to individuals spending more time outdoors and exhibiting a greater inclination toward using natural ventilation in buildings.^36^, ^37^ Such behaviors could potentially enhance the effects of ambient air pollution.

One limitation of the current study was that the seasonal influenza data were collected from surveillance sentinel hospitals, and values varied between years, which could have negatively affected the results. In addition, we interpolated daily incidence rates from the weekly data, which may artificially reduce variability and lead to underestimated effects. Thus, where available, using daily positive ILI rate data would likely prove advantageous. Finally, some climate factors, such as solar radiation and absolute humidity, were not included in the time‐series models due to limited data. These factors may have an impact on the concentration of ambient pollutants and the R_t_.

In conclusion, by using influenza transmissibility rather than reported ILI cases or incidence rates, we found that most ambient pollutants (i.e., PM_2.5_, PM_10_, NO_2_, SO_2_, and CO) were significantly associated with increased influenza transmissibility. The association between ambient pollutants and influenza transmissibility remained significant after adjusting the effect of depletion in susceptibles.

## AUTHOR CONTRIBUTIONS

**Jiao Yang**: Methodology; software; investigation; writing—original draft. **Guohui Fan**: Writing—original draft; methodology; writing—review and editing. **Li Zhang**: Methodology; software; investigation. **Ting Zhang**: Methodology; writing—review and editing. **Yunshao Xu**: Methodology. **Luzhao Feng**: Conceptualization; supervision; validation; funding acquisition; writing—original draft. **Weizhong Yang**: Conceptualization; methodology; funding acquisition.

## CONFLICT OF INTEREST STATEMENT

The authors declare that they have no competing interests.

### PEER REVIEW

The peer review history for this article is available at https://www.webofscience.com/api/gateway/wos/peer-review/10.1111/irv.13177.

## ETHICS APPROVAL STATEMENT

As this study is based on a secondary dataset without any identifying individual information, ethical approval was not needed. This study used a secondary dataset, and thus patient consents are not applicable.

## Supporting information


**Table S1.** Percentage of the variance of the instantaneous reproduction number (Rt) explained by individual drivers of influenza. The results based on the best lag (i.e. the lag for which the model has the largest R^2^ value).
**Table S2.** Percentage of the variance of the adjusted instantaneous reproduction number (R_t_) explained by individual drivers of influenza. The results based on the best lag (i.e. the lag for which the model has the largest R^2^ value).

## Data Availability

Because of the potentially sensitive information included, the original dataset is not public and is available from the corresponding author upon reasonable request.
