# Supplementary material for: The association between ambient pollutants and influenza transmissibility: A nationwide study involving 30 provinces in China
Source: Influenza Other Respir Viruses. 2023 Jul 23;17(7):e13177. doi: 10.1111/irv.13177 (PMC10363796; doi:10.1111/irv.13177)
Supplement: Supplementary file 1 — Table S1. Percentage of the variance of the instantaneous reproduction number (Rt) explained by individual drivers of influenza. The results based on the best lag (i.e. the lag for which the model has the largest R2 value). Table S2. Percentage of the variance of the adjusted instantaneous reproduction number (Rt) explained by individual drivers of influenza. The results based on the best lag (i.e. the lag for which the model has the largest R2 value). [file IRV-17-e13177-s001.docx]

**The association between ambient pollutants and influenza transmissibility: a nationwide study involving 30 provinces in China.**

Jiao Yang^1^, Guo-hui Fan^1,2^, Li Zhang^3^, Ting Zhang^1^, Yunshao Xu^1^, Luzhao Feng ^1.*^, Weizhong Yang^1.*^

^1^School of Population Medicine and Public Health, Chinese Academy of Medical Sciences & Peking Union Medical College, Beijing, China

^2^National Center for Respiratory Medicine; National Clinical Research Center for Respiratory Diseases; Institute of Respiratory Medicine, Chinese Academy of Medical Sciences & Peking Union Medical College; Department of Clinical research and Data management, Center of Respiratory Medicine, China-Japan Friendship Hospital, Beijing, Beijing, China

^3^School of Life Course and Population Sciences, King’s College London, London, United Kingdom

^*^ Corresponding author: School of Population Medicine and Public Health, Chinese Academy of Medical Sciences & Peking Union Medical College, No.31 BeiJiGe 3 Alley, DongCheng District，Beijing,10005, China

E-mail address: yangweizhong@cams.cn (W-Z.Yang), fengluzhao@cams.cn (L-Z.Feng).

**CONTENTS**

**1. Estimation of Transmissibility**

**2. Derivation of adjusted**$\boldsymbol{R}_{\boldsymbol{t}}$

**3. Supplementary tables**

*3.1Table S1.*

*3.2Table S2.*

**4. References**

1. Estimation of Transmissibility

According to the Bayesian framework applied to the branching process model proposed by Cori et al.,^1^, the weekly instantaneous reproduction number $R_{t}$ was estimated, which is an extension of Fraser method^2^. Fraser proposed that the renewal estimation equation for the $R_{t}$ of an epidemic could be as

The renewal estimation equation for the$R_{t}$ of an epidemic could be as follow:

$$R_{t}=\frac{I_{t}}{\sum_{s=0}^{m} w_{s}I_{t-s}} (1.1)$$

where $I_{t}$ refers to the number of reported cases (here, incidence rate times a constant) between time $t$and time $t+1$ and $w_{s}$ refers to the generation time distribution, such that $\sum_{s=0}^{m} w_{s}=1$. The expected incidence at time $t$ is Poisson distributed with a mean ($R_{t}\sum_{s=0}^{m} w_{s}I_{t-s}$). The transmissibility is assumed to be constant over the time period [$t-\tau, t$] and measured by the $R_{[t-\tau,t]}$; then, the likelihood of $I_{t-\tau}, \ldots\ldots\ldots,I_{t}$ given the reproduction number $R_{\left[ t-\tau,t \right]}$ and $I_{0}, \ldots\ldots\ldots,I_{t-\tau-1}$ is as follows:

$${P(I}_{t-\tau}, \ldots\ldots\ldots,I_{t}\left| I_{0}, \ldots\ldots\ldots,I_{t-\tau-1}, w,R_{\left[ t-\tau,t \right]}) \right.=\prod_{s=t-\tau}^{t} \frac{{{e^{-R_{\left[ t-\tau,t \right]}\Lambda_{s}} (R}_{\left[ t-\tau,t \right]}\Lambda_{s})}^{I_{s}}}{I_{s}!} (1.2)$$

Where, $\Lambda_{s}=\sum_{s=0}^{m} w_{s}I_{t-s}$. The generation time distribution is a gamma distribution with a mean of 2.6 days (SD=1.5 d)^3^ and is assumed to be constant throughout an epidemic. A Bayesian framework with a Gamma-distributed prior with parameters (a, b) was developed for $R_{\left[ t-\tau,t \right]}$ and the posterior joint distribution of $R_{\left[ t-\tau,t \right]}$ can be derived as proportional to

$${{R_{\left[ t-\tau,t \right]}(posterior)=R}_{\left[ t-\tau,t \right]}}^{a+\sum_{s=t-\tau}^{t} I_{s}-1} e^{-R_{\left[ t-\tau,t \right]}(\sum_{s=t-\tau}^{t} \Lambda_{s}+\frac{1}{b})} \prod_{s=t-\tau}^{t} \frac{{\Lambda_{s}}^{I_{s}}}{I_{s}!} (1.3)$$

Equation (3) indicates that the posterior distribution of $R_{\left[ t-\tau,t \right]}$is a gamma distribution with the parameters$(a+\sum_{s=t-\tau}^{t} I_{s},{(\sum_{s=t-\tau}^{t} \Lambda_{s}+\frac{1}{b})}^{-1})$.

**3. Derivation of adjusted**$\boldsymbol{R}_{\boldsymbol{t}}$

We evaluated the adjusted $R_{t}$ by eliminating the effect of depletion of susceptibles from$R_{t}$ following the method described by Ali et al^4^. $R_{t}$ is a function of basic reproduction number ($R_{0}$) and depletion of susceptibles ($S_{t}$), therefore decreasing in trend as epidemic progress.

We first fit a model $ln\left( R_{t} \right)=\ln\left( R_{0}S_{0} \right)+\xi h_{t}$ to the estimated$R_{t}$ for each epidemic separately. $R_{0}$ is the basic reproduction number. $S_{0j}$ is initial susceptible proportion for epidemic $j$, obtained from Wang and colleagues’ study^5^.$h_{ij}$ is the observed cumulative incidence up to $(i-1)$th day of $j$th epidemic.$h_{ij}=\sum_{x}^{i-1} I_{xj}$, where $I_{xj}$ denotes the incidence in day $x$ of season 𝑗^5,6^. where $\xi={-c_{i}/S}_{0j}$ with constant$c_{i}$ (here$,c_{i}=1$). The adjusted $R_{t}$ was defined as the residual of the fit, thus the measure of adjusted $R_{t}$ is free from the effects of depletion of susceptibles and assumed to be driven by the inter-epidemic effects and the extrinsic drivers.

Table S1. Percentage of the variance of the instantaneous reproduction number (Rt) explained by individual drivers of influenza. The results based on the best lag (i.e. the lag for which the model has the largest R^2^ value).

| **Province** | **O_3_ (**$ug/m^{3}$) | **PM_2.5_ (**$ug/m^{3}$) | **PM_10_ (**$ug/m^{3}$) | **SO_2_ (**$ug/m^{3}$) | **CO (**$mg/m^{3}$) | **NO_2_ (**$ug/m^{3}$) | **Temperature (℃）** | **Relative humidity (%)** | **absolute humidity (g/m^3^)** |
| --- | --- | --- | --- | --- | --- | --- | --- | --- | --- |
| **Northern** |  |  |  |  |  |  |  |  |  |
| Beijing | 0.08 | 0.02 | 0.01 | 0.01 | 0.03 | 0.05 | 0.18 | 0.08 | 0.16 |
| Tianjin | 0.09 | 0.00^†, ‡^ | 0.00^†, ‡^ | 0.00^†, ‡^ | 0.01 | 0.01 | 0.03 | 0.01 | 0.00^†, ‡^ |
| Hebei | 0.16 | 0.00^†, ‡^ | 0.00^†, ‡^ | 0.01 | 0.01 | 0.04 | 0.06 | 0.00^†, ‡^ | 0.02 |
| Shanxi | 0.13 | 0.02 | 0.03 | 0.01 | 0.01 | 0.07 | 0.02 | 0.01 | 0.02 |
| Inner Mongolia | 0.12 | 0.01 | 0.01 | 0.01 | 0.03 | 0.05 | 0.01 | 0.01 | 0.01 |
| **Northeast** |  |  |  |  |  |  |  |  |  |
| Liaoning | 0.12 | 0.00^†, ‡^ | 0.00^†, ‡^ | 0.02 | 0.01 | 0.02 | 0.02 | 0.03 | 0.01 |
| Jilin | 0.06 | 0.02 | 0.01 | 0.03 | 0.02 | 0.01 | 0.05 | 0.03 | 0.07 |
| Heilongjiang | 0.19 | 0.01 | 0.00^†, ‡^ | 0.02 | 0.02 | 0.03 | 0.1 | 0.11 | 0.09 |
| **Northwest** |  |  |  |  |  |  |  |  |  |
| Shaanxi | 0.22 | 0.01 | 0.01 | 0.03 | 0.03 | 0.06 | 0.07 | 0.01 | 0.02 |
| Gansu | 0.12 | 0.01 | 0.01 | 0.03 | 0.03 | 0.06 | 0.03 | 0.02 | 0.07 |
| Qinghai | 0.1 | 0.03 | 0.00^†, ‡^ | 0.03 | 0.06 | 0.15 | 0.05 | 0.02 | 0.03 |
| Ningxia | 0.05 | 0.00^†, ‡^ | 0.02 | 0.00^†, ‡^ | 0.00^†, ‡^ | 0.01 | 0.02 | 0.06 | 0.04 |
| Xinjiang | 0.04 | 0.02 | 0.01 | 0.03 | 0.01 | 0.03 |  |  |  |
| **Eastern** |  |  |  |  |  |  |  |  |  |
| Shanghai | 0.06 | 0.01 | 0.02 | 0.01 | 0.01 | 0.05 | 0.00^†, ‡^ | 0.01 | 0.00^†, ‡^ |
| Jiangsu | 0.11 | 0.01 | 0.01 | 0.01 | 0.01 | 0.04 | 0.01 | 0.00^†, ‡^ | 0.00^†, ‡^ |
| Zhejiang | 0.08 | 0.03 | 0.03 | 0.02 | 0.03 | 0.07 | 0.00^†, ‡^ | 0.00^†, ‡^ | 0.00^†, ‡^ |
| Anhui | 0.1 | 0.02 | 0.01 | 0.01 | 0.01 | 0.02 | 0.01 | 0.00^†, ‡^ | 0.00^†, ‡^ |
| Fujian | 0.02 | 0 | 0.01 | 0.01 | 0.00^†, ‡^ | 0.02 | 0.01 | 0.01 | 0.01 |
| Jiangxi | 0.03 | 0.02 | 0.02 | 0.02 | 0.03 | 0.03 | 0.01 | 0.00^†, ‡^ | 0.01 |
| Shandong | 0.17 | 0.02 | 0.02 | 0.04 | 0.04 | 0.08 | 0.02 | 0.01 | 0.01 |
| **Central** |  |  |  |  |  |  |  |  |  |
| Henan | 0.03 | 0.04 | 0.03 | 0.01 | 0.04 | 0.02 | 0.01 | 0.01 | 0.00^†, ‡^ |
| Hubei | 0.03 | 0.01 | 0.00^†, ‡^ | 0.01 | 0.01 | 0.01 | 0 | 0.01 | 0.01 |
| Hunan | 0.03 | 0.04 | 0.03 | 0.01 | 0.04 | 0.02 | 0.01 | 0.01 | 0.00^†, ‡^ |
| **Southern** |  |  |  |  |  |  |  |  |  |
| Guangdong | 0.03 | 0.01 | 0.01 | 0.02 | 0.02 | 0.03 | 0.00^†, ‡^ | 0.01 | 0.00^†, ‡^ |
| Guangxi | 0.03 | 0.01 | 0.01 | 0.03 | 0.03 | 0.06 | 0.05 | 0.01 | 0.05 |
| Hainan | 0.01 | 0.00^†, ‡^ | 0.00^†, ‡^ | 0.00^†, ‡^ | 0.03 | 0.00^†, ‡^ | 0.00^†, ‡^ | 0.02 | 0.00^†, ‡^ |
| **Southwest** |  |  |  |  |  |  |  |  |  |
| Chongqing | 0.05 | 0.01 | 0.00^†, ‡^ | 0.00^†, ‡^ | 0.00^†, ‡^ | 0.00^†, ‡^ | 0.00^†, ‡^ | 0.05 | 0.00^†, ‡^ |
| Sichuan | 0.01 | 0.00^†, ‡^ | 0.00^†, ‡^ | 0.00^†, ‡^ | 0.00^†, ‡^ | 0.00^†, ‡^ | 0.00^†, ‡^ | 0.01 | 0.01 |
| Guizhou | 0.02 | 0.00^†, ‡^ | 0.00^†, ‡^ | 0.00^†, ‡^ | 0.01 | 0.00^†, ‡^ | 0.01 | 0.03 | 0.00^†, ‡^ |
| Yunnan | 0.03 | 0.00^†, ‡^ | 0.00^†, ‡^ | 0.03 | 0.02^†^ | 0.01 | 0.01 | 0.01 | 0.00^†, ‡^ |

† refers to the situation where the p-value in the log-linear regression is greater than or equal to 0.05.

‡ refers to the scenario where the p-value in the permutation test is greater than or equal to 0.05.

Table S2. Percentage of the variance of the adjusted instantaneous reproduction number (R_t_) explained by individual drivers of influenza. The results based on the best lag (i.e. the lag for which the model has the largest R^2^ value).

| **Province** | **O_3_ (**$ug/m^{3}$) | **PM_2.5_ (**$ug/m^{3}$) | **PM_10_ (**$ug/m^{3}$) | **SO_2_ (**$ug/m^{3}$) | **CO (**$mg/m^{3}$) | **NO_2_ (**$ug/m^{3}$) | **Temperature (℃）** | **Relative humidity (%)** | **absolute humidity (g/m^3^)** |
| --- | --- | --- | --- | --- | --- | --- | --- | --- | --- |
| **Northern** |  |  |  |  |  |  |  |  |  |
| Beijing | 0.08 | 0.02 | 0.02 | 0.01 | 0.04 | 0.05 | 0.17 | 0.07 | 0.16 |
| Tianjin | 0.29 | 0.02 | 0.01 | 0.04 | 0.04 | 0.06 | 0.11 | 0.02 | 0.03 |
| Hebei | 0.36 | 0.03 | 0.01 | 0.03 | 0.07 | 0.11 | 0.19 | 0.01 | 0.03 |
| Shanxi | 0.28 | 0.06 | 0.04 | 0.13 | 0.1 | 0.08 | 0.09 | 0.05 | 0.02 |
| Inner Mongolia | 0.34 | 0.05 | 0.00 ^†, ‡^ | 0.09 | 0.13 | 0.1 | 0.11 | 0.05 | 0.08 |
| **Northeast** |  |  |  |  |  |  |  |  |  |
| Liaoning | 0.33 | 0.02 | 0.01 | 0.11 | 0.07 | 0.05 | 0.16 | 0.07 | 0.07 |
| Jilin | 0.19 | 0.01 | 0.01 | 0.03 | 0.01 | 0.02 | 0.09 | 0.14 | 0.04 |
| Heilongjiang | 0.34 | 0.02 | 0.00^†, ‡^ | 0.18 | 0.11 | 0.11 | 0.26 | 0.36 | 0.17 |
| **Northwest** |  |  |  |  |  |  |  |  |  |
| Shaanxi | 0.5 | 0.07 | 0.04 | 0.12 | 0.15 | 0.12 | 0.14 | 0.06 | 0.01 |
| Gansu | 0.43 | 0.07 | 0.01 | 0.18 | 0.19 | 0.19 | 0.17 | 0.02 | 0.21 |
| Qinghai | 0.47 | 0.12 | 0.04 | 0.21 | 0.27 | 0.46 | 0.26 | 0.00^†, ‡^ | 0.25 |
| Ningxia | 0.09 | 0.00^†, ‡^ | 0.01 | 0.01 | 0.01 | 0.02 | 0.01 | 0.08 | 0.05 |
| Xinjiang | 0.27 | 0.00^†, ‡^ | 0.00^†, ‡^ | 0.00 ^†, ‡^ | 0.01 | 0.00^†, ‡^ | 0.03 | 0.1 | 0.01 |
| **Eastern** |  |  |  |  |  |  |  |  |  |
| Shanghai | 0.08 | 0.01 | 0.01 | 0.02 | 0.02 | 0.03 | 0.01 | 0.00^†, ‡^ | 0.02 |
| Jiangsu | 0.12 | 0.01 | 0.01 | 0.09 | 0.04 | 0 | 0.01 | 0.04 | 0.05 |
| Zhejiang | 0.12 | 0.03 | 0.03 | 0.09 | 0.06 | 0.04 | 0.01 | 0.00^†, ‡^ | 0.02 |
| Anhui | 0.29 | 0.02 | 0.01 | 0.09 | 0.05 | 0.01 | 0.01 | 0.02 | 0.02 |
| Fujian | 0.07 | 0.03 | 0.05 | 0.15 | 0.08 | 0.11 | 0.05 | 0.02 | 0.05 |
| Jiangxi | 0.04 | 0.05 | 0.04 | 0.08 | 0.07 | 0.06 | 0.01 | 0.00 ^†, ‡^ | 0.01 |
| Shandong | 0.37 | 0.08 | 0.07 | 0.24 | 0.2 | 0.16 | 0.09 | 0.02 | 0.01 |
| **Central** |  |  |  |  |  |  |  |  |  |
| Henan | 0.25 | 0.02 | 0.01 | 0.05 | 0.08 | 0.09 | 0.00^†, ‡^ | 0.08 | 0.05 |
| Hubei | 0.05 | 0.02 | 0.01 | 0.06 | 0.04 | 0.00^†, ‡^ | 0.02 | 0.02 | 0.04 |
| Hunan | 0.03 | 0.01 | 0.01 | 0.12 | 0.09 | 0.00^†, ‡^ | 0.01 | 0.06 | 0.05 |
| **Southern** |  |  |  |  |  |  |  |  |  |
| Guangdong | 0.02 | 0.06 | 0.07 | 0.19 | 0.13 | 0.07 | 0.03 | 0.00^†, ‡^ | 0.02 |
| Guangxi | 0.00^†, ‡^ | 0.03 | 0.04 | 0.17 | 0.15 | 0.09 | 0.05 | 0.01 | 0.06 |
| Hainan | 0.02 | 0.05 | 0.04 | 0.04 | 0.16 | 0.02 | 0.08 | 0.04 | 0.05 |
| **Southwest** |  |  |  |  |  |  |  |  |  |
| Chongqing | 0.17 | 0.01 | 0.02 | 0.09 | 0.04 | 0.01 | 0.02 | 0.05 | 0.00^†, ‡^ |
| Sichuan | 0.00^†, ‡^ | 0.02 | 0.02 | 0.01 | 0.01 | 0.01 | 0.03 | 0.05 | 0.06 |
| Guizhou | 0.06 | 0.01 | 0.01 | 0.03 | 0.00^†, ‡^ | 0.01 | 0.00^†, ‡^ | 0.07 | 0.05 |
| Yunnan | 0.17 | 0.01 | 0.02 | 0.09 | 0.04 | 0.01 | 0.02 | 0.05 | 0 |

† refers to the situation where the p-value in the log-linear regression is greater than or equal to 0.05.

‡ refers to the scenario where the p-value in the permutation test is greater than or equal to 0.05.

**4. References**

1. Cori A, Ferguson NM, Fraser C, Cauchemez S. A new framework and software to estimate time-varying reproduction numbers during epidemics. *Am J Epidemiol.* 2013;178(9):1505-1512.

2. Fraser C. Estimating individual and household reproduction numbers in an emerging epidemic. *PLoS One.* 2007;2(8):e758.

3. Cauchemez S, Donnelly CA, Reed C, et al. Household transmission of 2009 pandemic influenza A (H1N1) virus in the United States. *The New England journal of medicine.* 2009;361(27):2619-2627.

4. Ali ST, Cowling BJ, Wong JY, et al. Influenza seasonality and its environmental driving factors in mainland China and Hong Kong. *Sci Total Environ.* 2022;818:151724.

5. Wang Q, Yue N, Zheng M, et al. Influenza vaccination coverage of population and the factors influencing influenza vaccination in mainland China: A meta-analysis. *Vaccine.* 2018;36(48):7262-7269.

6. te Beest DE, van Boven M, Hooiveld M, van den Dool C, Wallinga J. Driving factors of influenza transmission in the Netherlands. *Am J Epidemiol.* 2013;178(9):1469-1477.
